# The Environmental Impact Assessment of Sanitation Projects in Chile: Overview and Improvement Opportunities Focused on Follow-Ups

**DOI:** 10.3390/ijerph19073964

**Published:** 2022-03-26

**Authors:** Dante Rodríguez-Luna, Francisco Javier Alcalá, Francisco Encina-Montoya, Nuria Vela

**Affiliations:** 1 Department of Civil Engineering, Catholic University of Murcia, 30107 Murcia, Spain; derodriguez@alu.ucam.edu; 2 Facultad de Ingeniería, Instituto de Ciencias Químicas Aplicadas, Universidad Autónoma de Chile, Santiago 8910060, Chile; fjalcala@eeza.csic.es; 3 Departamento de Desertificación y Geo-Ecología, Estación Experimental de Zonas Áridas (EEZA-CSIC), 04120 Almeria, Spain; 4 Nucleus of Environmental Studies, Catholic University of Temuco, Temuco 4781312, Chile; fencina@uct.cl; 5 Applied Technology Group to Environmental Health, Faculty of Health Science, Catholic University of Murcia, 30107 Murcia, Spain

**Keywords:** environmental impact assessment, sanitation projects, human health, principal coordinate analysis, Chile

## Abstract

The Environmental Impact Assessment (EIA) is a legal and administrative tool aimed to identify, predict, and interpret the impact of a project or activity on the environment and human health. The EIA also evaluates the accuracy of the predictions and audits the effectiveness of the established preventive measures. Regarding the sanitation sector, efficiency of wastewater treatments and sanitation networks determine the pollutant level of the discharged liquid effluents and the subsequent impact on the environment and human health. This problematic makes necessary to assess how proper the regulatory follow-ups of sanitation projects is. This paper evaluates the performance of the Chilean EIA System concerning to sanitation projects. Taking into account that the more restrictive Environmental Impact Study (EIS) and more permissive Environmental Impact Declaration (EID) are the ways for projects’ entry to the EIA System in Chile, 5336 sanitation projects submitted to EIA between 1994 and 2019 were complied. A representative sample of 76 projects (15 entered as EIS and 61 as EID) was analyzed by using a principal coordinate analysis (PCoA) through 14 selected performance indicators. Observed weaknesses have led to propose improvement opportunities of the EIA focused on the follow-ups after the environmental license is obtained, such as creation of a simplified sanctioning procedure, decentralization of decision-making, deadline establishment in each stage, and unified direct link for each project. These proposals seek to improve the effectiveness of monitoring and possible sanctions to early identify impacts of sanitation projects on the environment and human health. This paper introduces a robust methodology for evaluation criteria focused on the follow-ups analysis, which can be used in other countries that consider respectful sanitation projects have direct social and environmental benefits leading to long-term indirect cultural and economic values.

## 1. Introduction

Water scarcity affects every continent and is one of the main global challenges. Water consumption has increased at twice the rate of population growth and, although there is enough drinking water on the planet, it is unevenly distributed, wasted, polluted in many cases, and often unsustainably managed. The problem of urban, agricultural, and industrial supply is exacerbated by the fact that a large fraction of surface watercourses and groundwater bodies are polluted by discharges of untreated or deficiently treated liquid effluents generated by human activity. This reduces the availability of drinking water and introduces the necessity of using wastewater treatment technology, which is not always viable or regulated enough in all countries.

Discharge of untreated or deficiently treated urban wastewater (including domestic, industrial and sanitary effluents) to water bodies (lakes, rivers, aquifers, sea) causes negative effects on the environment and human health. The indirect consequence is biodiversity loss of aquatic systems, and increasing economic and social gaps that hamper the sustainable development of modern societies [1,2,3]. Wastewater sanitation systems, which include collection and distribution pipeline networks and treatment plants, are essential for the sustainable social and economic development of the territories. In 2015, sanitation was recognized essential by the United Nations General Assembly. Some strategies were agreed in the Millennium Development Goals through the Sustainable Development Goals (SDG), specifically the goal 6 “Ensure availability and sustainable management of water and sanitation for all” [4,5,6]. Unfortunately, the SDGs are not being met. In fact, more than half of the world population—around 4.2 billion people—do not have access to efficient sanitation services, leaving untreated or deficiently treated solid and liquid human waste [5].

In Chile, Decreto Supremo N° 90—Norma de emisión para la regulación de contaminantes asociados a las descargas de residuos líquidos a aguas marinas y continentales superficiales (Supreme Decree for emission standard of liquid water discharges to marine and continental surface waters) regulates wastewater discharge, including flow rates and quality standards [7], whereas Norma Chilena 1333—Requisitos de calidad de agua para diferentes usos (Chilean Regulation for water quality requirements for different usages) determines the quality standards for water use for urban supply, irrigation, recreation, and aquatic life [8]. Currently, the water supply coverage of urban areas is 99.9%, sewerage is 97.3%, and wastewater recovery is 99.9%. The existing 301 wastewater treatment plants use preferably secondary techniques (174 activated sludge, 57 aerated lagoons, 13 primary systems plus disinfection, 9 stabilization ponds, 7 sequential batch reactors, 4 oxidation ditches, 3 vermifilter, and 1 biofilter) and other primary ones subordinately; no tertiary or more efficient treatments are used [9]. As deduced, Chile still has a long way ahead in wastewater treatment and reuse, since that 96% of the secondary-treated wastewater is directly discharged to continental watercourses and the totality of the primary-treated wastewater is discharged to the sea through 33 submarine emissaries [10]. In short, the absence of a specific urban wastewater reuse regulation puts the environment and human health at risk, which is not understandable taking the water scarcity of the Chilean northern regions into account [11].

During the second half of the twentieth century, the states have enacted environmental protection instruments to cope with this increasing problem. In 1970, the United States of America (USA) through the National Environmental Policy Act incorporated the Environmental Impact Assessment (EIA) as a tool for environmental management and protection. From the 1970s onward, different countries such as Australia, Canada, Sweden, New Zealand, and the European Union (EU) states have enacted similar environmental instruments. In 1992, the United Nations Conference on the Environment held in Rio de Janeiro recognized the EIA as an instrument to reduce the negative effects of sanitation projects on the environment and human health [12,13]. In the EU, the Directive 2011/92/UE of 1992 [14] included the first EIA regulation and the Directive 2014/52/EU of 2014 [15] introduced additional requirements concerning to human health [16,17].

In Chile, Law 19,300 of Bases Generales del Medio Ambiente (General Environmental Bases) enacted in 1994, recognized the EIA as the decision-making administrate tool to identify, predict, and interpret the environmental impact of projects [18]. In 2010, the creation of the Ministry of the Environment included the Servicio de Evaluación Ambiental (Environmental Assessment Service, EAS) and Superintendencia del Medio Ambiente (Superintendency of the Environment, SE) to implement the EIA System. The EAS administers the EIA, and SE supervises the environmental licenses [19]. Since 1997, Chile has included three regulatory periods dictated by Decreto Supremo N° 30/1997 (Supreme Decree, SD30) since 1997 [20], Decreto Supremo N° 95/2001 (Supreme Decree, SD95) since 2001 [21], and Decreto Supremo N° 40/2012 (Supreme Decree, SD40) since 2012 [22]. The Chilean regulation establishes two ways for the projects’ entry as Estudio de Impacto Ambiental (Environmental Impact Study, EIS) and Declaración de Impacto Ambiental (Environmental Impact Declaration, EID) [19,22]. An EID is a declaration of regulatory compliance that must demonstrate that a project (or any of its components) does not generate significant impacts on the environment and human health. An EIS is devoted to projects with evident impacts on the environment and human health, which will be subjected to mitigation measures, compensation or repair to comply with environmental regulations. Another difference is that citizen participation is mandatory in the EIS, whereas in the EID it is only possible when the projects have environmental charges [22].

**Figure 1:**
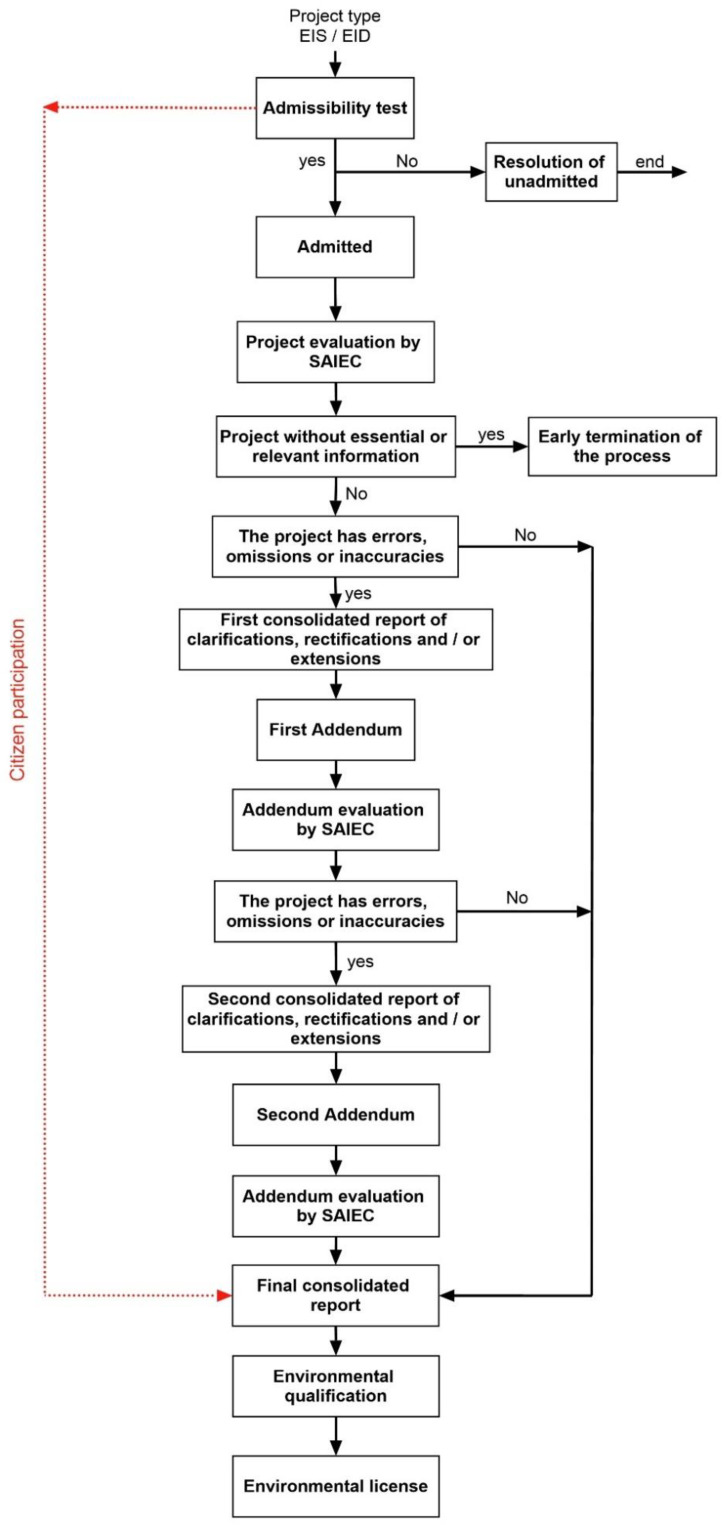
Flowchart of the Chilean EIA process, adapted from the EAS official website information. SAIEC—State Administration Institutions with Environmental Competence.

This environmental regulation considers human health as a determinant factor to rule out a sanitation project when it surpasses stablished standards. In the EID, the projects must rule out negative implications on human health due to quantity and quality of effluents and wastes to water bodies. In the EIS, the projects must consider mitigation, compensation, or repair measures when the activity implies significant affection on the environment and human health; the cases with low or null affection do not require special treatment [19,22]. The EAS manages the EIA process whereas the State Administration Institutions with Environmental Competence (SAIEC), including the Ministry of Health, evaluates whether the projects generate possible effects on human health. Figure 1 summarizes the Chilean EIA process. Table 1 describes those sanitation projects subjected to EIA, which are classified with the letter “o” in the Chilean legislation.

Regarding the EIA, the evaluation criteria described in the consulted scientific literature mostly focused on legislation, administration, and process [24,25,26,27]. In a recent assessment of the Chilean EIA, Rodríguez-Luna et al. [28] introduced the category “After the EIA” to include the public information of the process and its subsequent evaluation, supervision and sanction, and dispute resolution. This category was analyzed taking the relevance of the post-evaluation by citizens’ view into account, as previously proposed by the Presidential Advisory Commission for the evaluation of the EIA System [29].

**Figure 2:**
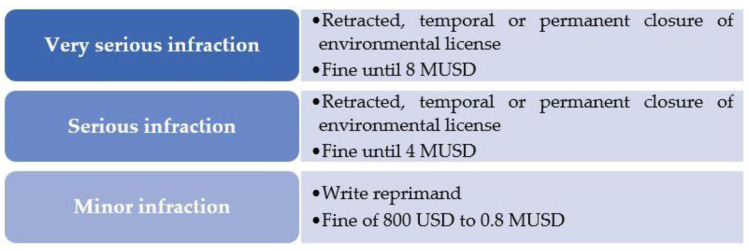
Ranking of sanctions attending to the infraction severity, adapted and updated from Methodological bases for the determination of environmental sanctions of SE [32]. Data source: (https://portal.sma.gob.cl/index.php/download/bases-metodologicas-para-la-determinacion-de-sanciones-ambientales-2017/) (accessed on 11 January 2022).

In Chile, SE is responsible of the regulatory follow-ups by auditing and sanctioning after the environmental license is obtained, which safeguards human health in the EIA process [28,30]. Law 20,417 details the three post-auditing types: (i) What is carried out directly for SE; (ii) what is performed through other public institutions; and (iii) what uses external institutions accredited and authorized by SE [19,30]. In relation to the environmental sanctions, Resolution 85 of SE [31] establishes a ranking attending to the infraction severity as minor, serious, and very serious [32], taking into account that the previous post-audit benefits the EIA performance [33] and the authority plays a critical role in the post-evaluation and follow-ups [34]. Figure 2 shows this raking of sanctions attending to the infraction severity.

This paper analyzes the performance of the Chilean EIA System to elucidate the impact of sanitation projects on the environment and human health. For this, (1) the state-of-art of the EIA of sanitation projects in Chile is reviewed, (2) adequate indicators to catalogue a representative sample of sanitation projects are identified and evaluated, and (3) some improvements to increase the EIA performance relative to standards focused on follow-ups of the sanitation sector are proposed.

## 2. Methods

### 2.1. Study Area

**Figure 3:**
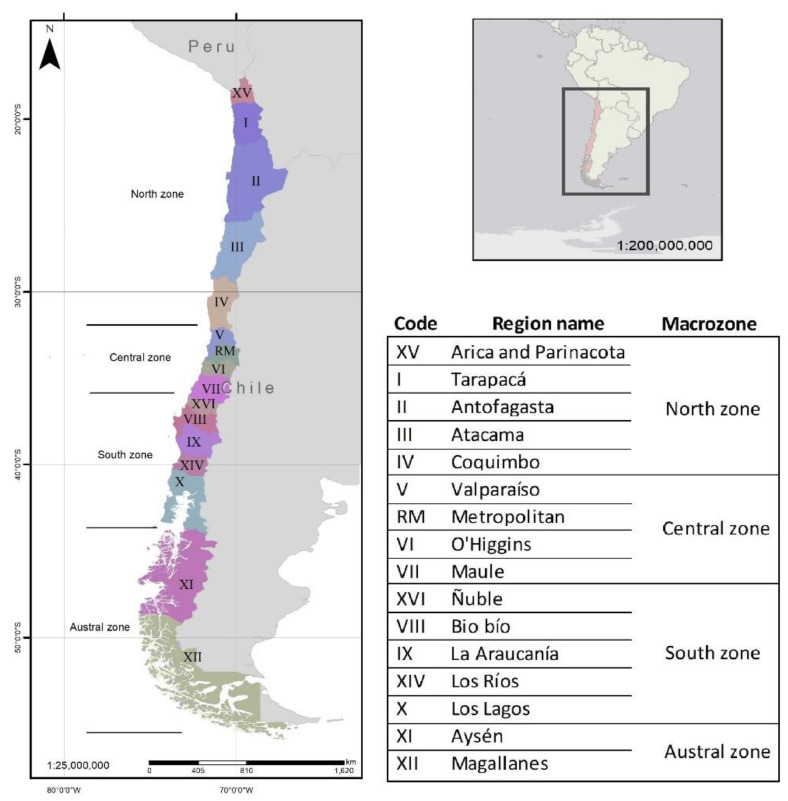
Macrozones and regions of Chile.

Chile extends between latitudes 17°29′57″ S and 56°32′12″ S in South America, occupies an elongated fringe of 200-km width between the Pacific Ocean and the Andes Cordillera of around 4270 km^2^ of surface (the islands territories are excluded), and is divided into sixteen regions grouped into four macrozones (Figure 3). Chile had 17.5 million people censused in 2017, from which around 2.2 million are indigenous people according to the National Statistics Institute of Chile [35]. Figure 3 shows the division of macrozones and regions of Chile.

### 2.2. Data Source

Information concerning to all “type o” sanitation projects was compiled from the EAS official website (https://www.sea.gob.cl) (accessed on 12 January 2021). The compilation considered all possible status of the projects—here called project classification types—as approved, rejected, in evaluation, unadmitted, no rated, abandoned, desisted, and license expired [36,37,38].

### 2.3. Selection of Projects

Probability sampling methods are frequently used to determine the sample size [39,40]. In this paper, a proportional stratified sampling method [41] was used, taking into account that the sample represents finite population, and the statistical significance responds to an imposed confidence level of 90% and an absolute error less than 10% [42,43]. The used formulation was:

(1) $$\eta =\frac{Ν\;Z^{2}\;p\;q}{d^{2}\;\left(N-1\right)+Z^{2}\;p\;q}$$

where *η* = sample size, *d* = sample absolute error, *N* = population size, *p* = percentage of individuals with state or condition, *q* = percentage of individuals without state or condition, and *Z* = imposed confidence level. All variables and parameters are dimensionless.

The sample size (number of sanitation projects) was randomly selected over the category “approved” only. This selection excluded expressly the interregional projects. The 76 selected sanitation projects were catalogued by macrozone and region (Table 2).

### 2.4. Selection of Indicators

With the aim of identifying the most suitable indicators, the evaluation criteria originally proposed by Wood [24], Annandale [25], Ahmad and Wood [26], Khosravi et al. [27], and Rodríguez-Luna et al. [28,36] were reassessed, and the main post EIA weaknesses advised by the Presidential Advisory Commission for the EIA System evaluation, i.e., public information, and supervision/sanction for non-compliance were considered. The selection of indicators was focused on the categories “EIA process” and “After EIA” defined by Rodríguez-Luna et al. [28,36]. Table 3 summarizes the 14 selected indicators some being adapted for their correct application in a sample of projects. Indicators A, B, C, D, E, F, G, H, I, and N refer to the category “EIA process”, whereas indicators J, K, L and M refer to the category “After EIA”. As proposed by Arvidsson [44] and Fayers [45], a quantitative score for reliable comparisons of the selected projects is used through an ordinal scale for indicators A, B, K, L, M, and N, and a nominal scale (Yes = 2 or No = 1) for indicators C, D, E, F, G, H, I, and J.

### 2.5. Statistical Analysis

A principal coordinate analysis (PCoA) was implemented to identify the data structure [46], i.e., the observed patterns and relationships between sanitation projects (Table 2) attending to the selected indicators (Table 3). The PCoA (1) reduces dimensionality, finds similarities between objects and indicators, and preserves the relevant relationships’ information between the data sample; and (2) determines the data structure in a distance (or similarity) matrix, even when the dataset does not fulfil a normal distribution and the number of variables exceeds the sample size [47,48]. Initially, the variables were square-rooted transformed, the result was standardized by the total, and a similarity matrix combining indicators and sanitation projects based on Euclidean distance was created [48]. In order to compare the project types, an ANOVA analysis with permutational multivariate analysis of variance (Permanova) was implemented, and a hierarchical cluster analysis by using the Simprof test was performed. For the numerical and graphical analysis, the Primer 7 v7.0.13 program from PRIMER-e 2020^®^ was used.

## 3. Results and Discussion

### 3.1. The Sanitation Projects in the Chilean EIA System

**Figure 4:**
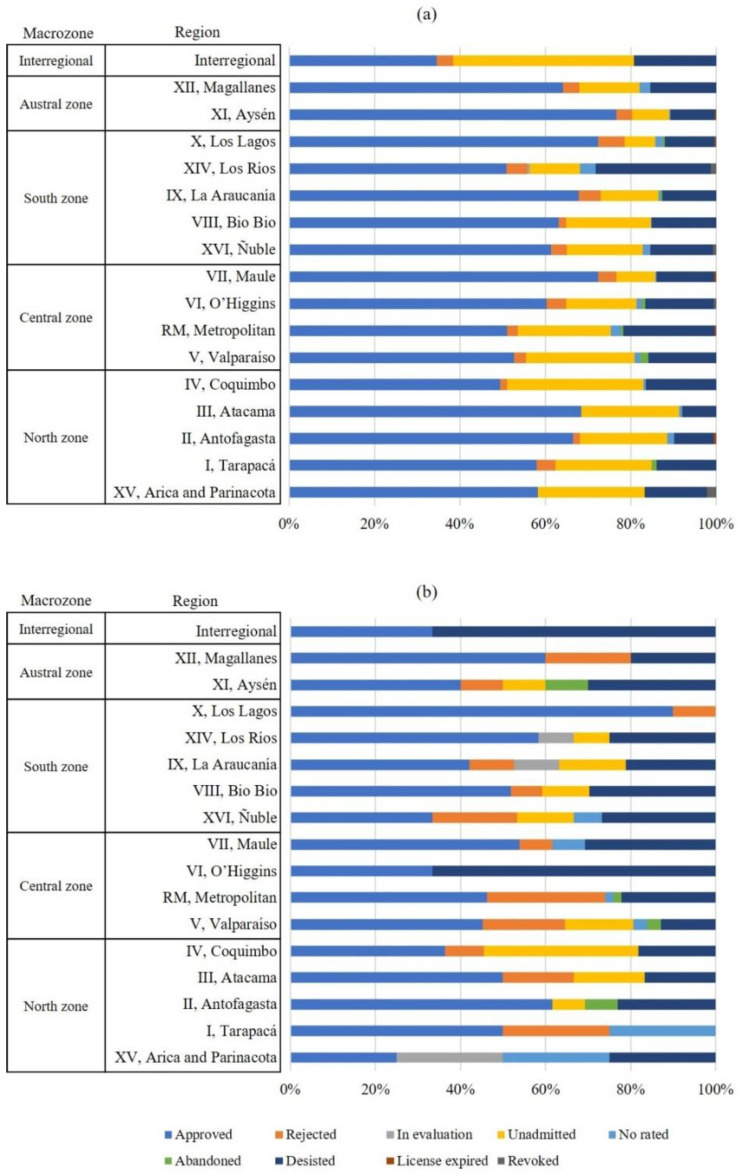
Clustered by regions, percentage of sanitation projects entered as EID (**a**) and EIS (**b**) to the Chilean EIA System. Interregional sanitation projects developed in two or more regions were omitted from the statistical analysis. Full information is included in Table A1 in Appendix A.

A total of 5336 sanitation projects were submitted to the Chilean EIA System between 1994 and 2019, both including 5087 projects entered as EID (95.3%) and 249 projects as EIS (4.7%). Figure 4 shows the percentage of sanitation projects entered as EIS and EID by region and status. All regions of the country computed sanitation projects. However, the regions Metropolitan (676 projects), Los Lagos (750 projects), and O’Higgins (676 projects) concentrated around 42% of the total submitted sanitation projects. The high percentages are positively correlated to the greatest population and industrial densities, thus making necessary sanitary solutions. Regarding the status of the projects, 62% were approved, 4.2% rejected, 0.1% in evaluation, 15.7% unadmitted, 1.3% not rated, 0.4% abandoned, 15.4% desisted, 0.1% expired, and less than 0.1% revoked to the environmental license. This status variability is similar to that formerly studied aquaculture projects submitted to the Chilean EIA System [36], with approved (68.9%), rejected (9.4%), and desisted (8.8%) projects. As expected, the Chilean EIA System does not discriminate among activities but among checkable qualities of the projects under the same evaluation criteria.

Regarding the sanitation projects that passed the EIA process, Los Lagos (90.0%), Magallanes (60.0%) and Los Ríos (58.3%) regions show the highest percentages. On the other hand, the lowest approval percentage corresponds to the Arica and Parinacota (25.0%) region. Regarding the rejected projects, Metropolitan (27.8%) and Tarapacá (25.0%) regions show the highest percentages. The causes are related to the non-compliance of regulations or impossibility of demonstrating that the projects do not generate impacts on the environment and human health. Arica and Parinacota, Antofagasta, O’Higgins, and Los Ríos regions do not register rejected projects, nor is there any interregional project rejected. Concerning to unadmitted projects, the high percentage relative to no rated projects in Coquimbo (36.4%) region stands out together with the score obtained in Arica and Parinacota (25.0%) and Tarapacá (25.0%) regions. Previous research pointed out different criteria for the declaration of inadmissibility of projects among regions [36]. Interregional and the O’Higgins region show the highest percentages (66.7%) of desisted projects.

Attending to the projects entered as EID, Aysén (76.7%), Los Lagos (72.4%), and Maule (72.4%) regions showed the highest approval percentages. Oppositely, interregional (34.6%) projects have the lowest approval percentages. Los Lagos (6.2%), La Araucanía (5.1%), and Los Ríos (4.9%) regions belonging to the South Macrozone show the highest percentages of rejected projects, which is explained by social conflicts in this macrozone. Regarding the projects not admitted, interregional projects (42.3%) show the highest percentage, followed by the Coquimbo region (31.8%). Los Ríos (27.0%) and Metropolitan (21.4%) regions show the highest percentages of desisted projects.

In comparative terms, the projects entered as EIS have an average approval percentage (47.7%) lower than those entered as EID (60.5%) and a higher average rejection percentage (Figure 4). The causes are the greater complexity for projects entry as EIS, including the greater required minimum content compared to the EID together with the additional difficulties related to the suitability of the proposed mitigation, compensation, and/or repair measures of significant environmental impacts [22,36]. In addition, the conflicts usually affect large projects, i.e., those entered as EIS.

### 3.2. Comparison of Sanitation Projects Entered as EIS and EID

**Figure 5:**
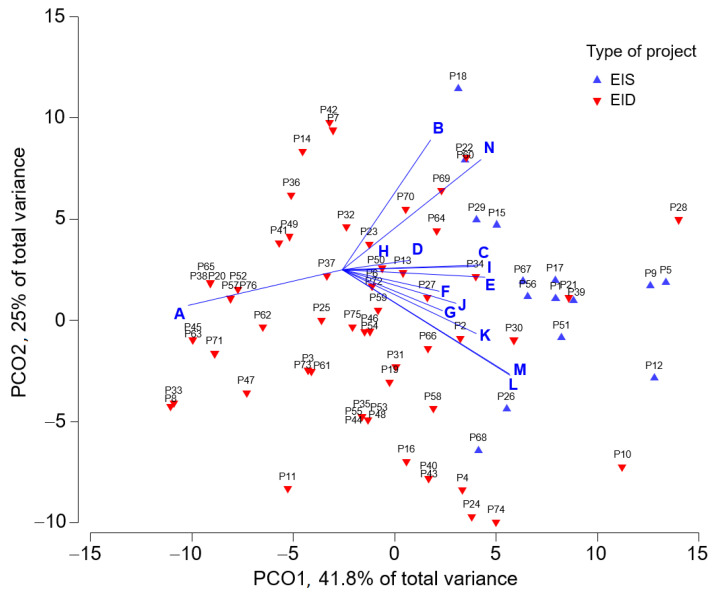
First factorial plane coordinates PCO1 and PCO2 of the PCoA performed on the 76 selected (sample) sanitation projects (15 entered as EIS and 61 as EID). Codes “A” throughout “N” identify the 14 selected indicators included in Table 2. Full information of selected sanitation projects is available in Table A1 in Appendix A.

Similarities and dissimilarities of sanitation projects entered as EIS and EID can be deduced from quantifiable statistical patterns and relationships obtained from the implemented PCoA. The PCoA included a sample of 76 sanitation projects compiled from the EAS website (15 EIS and 61 EID), and taken the singularity of the 14 selected indicators (Table 2) between projects entered as EIS and EID in account. Figure 5 shows the first factorial plane coordinates (PCO1 and PCO2) of the PCoA, which represents 66.8% of the total variance. This analysis identifies significant differences between EIS and EID, thus making possible to observe a homogeneous distribution of each EIS and EID statistical field. Table A1 in Appendix A includes the full information of the 76 selected sanitation projects, i.e., the PCO1 and PCO2 coordinates. Interpretation of results attending to the influence of the 14 selected indicators is below.

Processing Time (A). In EID, processing time was less than 90 days in 59% of projects and between 91 and 180 days in 31.3% of projects. In EIS, processing time of 60% of projects was between 91 and 180 working days, and the rest of projects over 181 working days. In general, projects entered as EID were quickly processed than those entered as EIS. This is identified in Figure 5 by the displacement of the indicator “A” toward the EID projects field. This agrees with the requirements established in the national regulation and other findings from former research focused on aquaculture projects in Chile [22,36]. For both projects entered as EID and EIS, the transmission time has decreased over time, especially in the current regulatory period SD40, which is identified as the strength of the EIA System.

Description of the Influence Area (B). In EID, the most relevant feature is that 26.2% of projects include general information only, 13.1% have moderately justified information, and 13.1% detail the influence area. In EIS, 40% of projects have moderately justified information and 33.3% detail the influence area. This better description in projects entered as EIS is identified in Figure 5 by slight displacement of the indicator B toward the EIS projects field. The improvement in the description of the influence area imposed by the successive, more restrictive regulatory periods (from SD30 to SD40) is also observed. The improving description of the influencing area is a consequence of the guides published by the EAS to describe land use, soil, flora, fauna, air quality, and human life systems. These guides relate the requirements that promoters must be incorporated in the projects [36]. The EIA also includes a guide to evaluate the effects on human health [49]. This criterion is quite important since a proper description of the influence area allows discarding or taking charge of the projects that could impact human health, renewable natural resources, human life systems, tourism and landscape values, and cultural heritage.

Methodology to Identify and Evaluate Environmental Impacts (C). All the projects entered as EIS used different methodologies to identify and evaluate the environmental impacts. The most commonly used methodology was a significance matrix [50] by means of qualitative criteria classified on an ordinal scale, specifically the Leopold matrix. This procedure was formerly also used by the authors to evaluate aquaculture projects in Chile [36]. Instead, no projects entered as EID used any specific methodology to assess the environmental impacts, since the current regulations do not impose this condition [22]. The EID only consider the justification that the project does not generate significant environmental impacts. Figure 5 shows the differences between the projects entered as EIS and EID, and how the indicator shifts significantly toward the EIS projects.

Use of International Regulations as a Reference (D). Reference international standards were used in 3.3% of projects entered as EID and in 20% of those entered as EIS, but only in the regulatory periods SD95 and SD40. The greater use in projects entered as EIS is due to the greater magnitude and complexity of the studies, which makes it necessary to use international standards in some cases.

Existence of Mitigation Measures (E). All the projects entered as EIS included mitigation measures, while the projects entered as EID differentiated additionally between environmental management measures and others such as mitigation, compensation and repair. In fact, a good conceptualization of the measures, with no confusion between them is observed.

Existence of Compensation Measures (F). This indicator is found in 33.3% of projects entered as EIS. In general, the projects consider mitigation measures as the first option followed by the compensation measures. However, no compensation measures are identified in the projects entered as EID.

Existence of Repair Measures (G). In contrast to that formerly reported in aquaculture projects [36], the sanitation projects are more aware to apply repair measures, despite that only 40% of projects entered as EIS implement this option. The projects entered as EID do not report this measure that implies a correct application of the different measures.

Identification of Contingency and Emergency Measures (H). About 80% of the projects consider this indicator. This figure increases up to 100% in the regulatory period SD40. There is no difference between projects entered as EID and EIS. This criterion has improved as the successive regulatory periods have been refined. The improvement of the results in the last regulatory period (SD40) shows how the better administrative performance by the EAS has positively impacted the technical content of the EIA reports.

Consultation and Participation (I). Regarding this indicator, the Article 94 of the SD40 establishes that only some types of projects generate environmental burdens, e.g., the “type o”, thus leading also the possibility of opening a citizen participation process in projects entered as EID [22,28]. All projects entered as EIS consider citizen participation, but only 35% of them include citizen observations. However, only 3.3% of projects entered as EID include a citizen participation process and none of them included citizen observations. The low early citizen participation is identified as weaknesses of the EIA System, which is an improvement opportunity in projects with environmental charges entered as EID and in all projects entered as EIS [29,36]. The addition of mandatory early participation would help to reduce the technical asymmetry between project promoters, citizens, and state agencies.

Appeal After Project Approval or Rejection (J). Around 93% of projects entered as EIS and 3.3% of those entered as EID use appeal options to complain non-conformities of the environmental license. The higher EIS percentage is related to the greater restrictions of the process. The bigger magnitude and complexity of negative externalities of the projects generate more social conflict and complaints. The projects’ promoters also disagree in relation to the conditions established by the authority for the projects’ approval. The opportunity to appeal after obtaining the environmental license is identified as strength of the EIA System.

Public Information After Obtaining the Environmental License (K). All projects entered as EIS and 75% of projects entered as EID include public information on environmental monitoring, audits, and/or sanctions after obtaining the environmental license. In 2010, the information available in the EAS website started to be published in the SE website. This difficulty to find information about some projects in the SE website, which is the same that the citizens find, was solved by searching through the file number. In comparison to other countries, this complex data accessing is identified as weakness of the EIA System [28], which is an improvement opportunity.

Post-auditing (L). Regarding the projects entered as EID, 21.3% of them present different compliance gaps with their environmental license and 44.3% present compliance. Regarding the projects entered as EIS, 73.3% of them present compliance gaps and 13.3% present compliance. Only 27.6% of projects are audited; all correspond to projects entered as EID. This relevant finding implies that the auditing priority is large projects. The audits carried out by the authority entail monitoring all requirements of the EIA process, so its implementation is essential to avoid impacts on human health during the project execution. The prioritization of large-scale projects is identified as weakness of the EIA System, so post-auditing is an improvement opportunity.

Punishment for Non-compliance (M). A total of 14.5% of projects present minor, 11.8% serious, and 2.6% very serious infractions. In addition, 39.5% of projects present compliance, most of them entered as EID. When analyzed by the typology, 73.3% of projects entered as EIS and 18% entered as EID present some type of infraction. These figures corroborate how auditing is mostly focused on those large projects generating more non-compliances with the environmental license. Increasing of the compliance is an improvement opportunity to reach higher national and international standards [51].

Investment (N). Around 46% of projects entered as EIS consider investments greater than 10 MUSD, while 74% of projects entered as EID consider investment under 10 MUSD. So, projects having large investment are generally entered as EIS.

In brief, the comparison of sanitation projects entered as EIS and EID has showed how the processing time (A) of the EID is significantly less than that of the EIS; the description of the influencing area (B) in most of the EIS is more detailed than in the EID; all the EIS used methodology to identify and assess environmental impacts (C) while neither EID used it; international regulations (D) are mainly used by the EIS; only the EIS applied good mitigation, compensation and repair measures (E, F and G); identification of contingency and emergency measures (H) have progressively been improved to reach the 100% of the EIS and EID in the last regulatory period (SD40); consultation and participation (I) is lower in the EID because the regulation imposes different conditions for EIS and EID; 93% of the projects entered as EIS mainly uses appeal after the approval or rejection (J); all the EIS and 75% of the EID have public information (K); the EIS shows higher percentages of post audit (L) than the EID; punishment for non-compliance (M) mostly affect the projects entered as EIS; and investment (N) less than 10 MUSD generally refers to the EID.

### 3.3. Improvement Opportunities of the EIA Process concerning to Sanitation Projects

In view of the trust that suitable follow-ups of the projects after obtaining its environmental license generated in the citizenry, post-evaluation was considered essential in the official reports of the presidential advisory commission for the EIA process evaluation [33]. Criteria such as *Public Information after obtaining the environmental license* (K), *Post-audit* (L), and *Sanction for non-compliance* (M) were considered weaknesses of the EIA System. Taking these weaknesses into account, three improvement opportunities concerning to the follow-ups of a sanitation project after obtaining its environmental license are proposed.

The first improvement is the creation of a streamlined sanctioning procedure aimed at minimizing sanction periods and compliance programs of small and medium-sized projects, which seeks to promote compliance with the project by committing its promoter to make improvements to keep onward. This is because all projects (large, medium-sized and small) have the same procedure, including the same deadline, but the authority prioritizes the large ones. Likewise, the compliance with the promotion is the instrument most used by promoters and must be limited to specific cases. This procedure makes it difficult for citizens to understand, since it often implies that those who have failed to comply with the obligations of the environmental license are exempt from fines [30].

The second improvement is decentralization of decision-making. The administrative structure of SE includes supervisors and heads of offices at the regional level while the annual inspection program is built regionally and the professionals in charge to validate decisions are centralized. This dichotomy undoubtedly affects autonomy and prioritization of regional problems because small projects (less complexed in general) compete with large projects (more complexed in general) during the resolution stage. This weakness makes necessary to establish deadlines for each stage, as happens with projects during the EIA process, where the regulation establishes the deadline of each stage. Previous studies also agreed that the high level of centralization affects decision-making of regional powers [30].

The third improvement is public information after obtaining the environmental license. This action should make it easy to access the project files between SE and EAS platforms [28]. Currently, each of the published project files can be independently accessed from the ES and EAS websites, but its search criteria are different and unfriendly. For instance, citizens cannot search by file number or environmental license number, as performed in this paper, because knowledge of how the structure of a file works is needed.

The above identified improvement opportunities are follow-ups related, so its implementation will improve information, supervision, and sanction stages of the projects after obtaining its environmental license. These actions will increase autonomy to the regions and the public trust in the EIA System.

## 4. Conclusions

In Chile, there is no specific urban wastewater reuse regulation, so direct discharge of effluents puts the environment and human health at risk. This is not understandable taking also into account the necessity for non-conventional water sources in the northern arid regions. In the past decades of the twentieth century, successive regulations have defined the Chilean EIA System as the environmental protection instrument to cope with this increasingly problematics. In this context, the assessment of the Chilean EIA System concerning to sanitation projects became an essential topic for research.

The Chilean EIA System includes the more restrictive EIS and more permissive EID ways for projects entry. In the period 1994–2019, a total of 5336 sanitation projects were entered to the EIA, 95.3% as EID and 4.7% as EIS. The statuses of the projects were 62% approved, 4.2% rejected, 0.1% under evaluation, 15.7% not admitted, 1.3% not qualified, 0.4% abandoned, 15.4% withdrawn, 0.1% expired, and 0.1% revoked. The implemented PCoA to measure the sample differences between projects entered to the EIA as EID and EIS showed homogeneous distributions of the EIS and EID statistical fields. The main statistical differences (indicators Processing Time; Description of the Influence Area; Methodology to Identify and Evaluate Environmental Impacts; Existence of Mitigation, Compensation and Repair Measures; Consultation and Participation; Post-auditing; and Punishment for Non-compliance) were weaknesses of the EIA System concerning sanitation projects, and were grouped into three improvement opportunities concerning the follow-up stages (*Creation of a simplified sanctioning procedure*, especially for small projects; *Decentralization of decision-making* with deadline establishment in each stage; *Public information after obtaining the environmental license* with possibility to consult projects though direct, unique link between the EAS and SE websites).

Incorporating these improvement opportunities in the regulatory follow-ups is a challenge to enhance the effectiveness of the post-auditing and punishment, thus increasing citizenry trust in the EIA System during the execution of sanitation projects. These improvement opportunities of the Chilean EIA System seek also to be a feasible methodology focused on the follow-ups analysis, which will be of assistance to other countries having similar environmental, social, and economic contexts.

## Figures and Tables

**Table 1:** Description of the sanitation projects subjected to EIA in Chile.

Letter	Description
o.1	Sewage systems for ≥10,000 inhabitants.
o.2	Sewage systems or stormwater evacuation facilities when they are interconnected for ≥10,000 inhabitants.
o.3	Drinking water systems that include uptaking and conveying water works, and intermediate processes to the user place for ≥10,000 inhabitants.
o.4	Wastewater treatment plants for ≥2500 inhabitants.
o.5	Treatment and/or disposal plants for solid waste from domestic and sanitary origin, transfer stations, and collection and classification centers serving ≥5000 inhabitants.
o.6	Submarine emissaries.
o.7	Treatment and/or disposal plants for liquid industrial waste, which meet one of the following conditions at least: (o.7.1) Include stabilization ponds. (o.7.2) Effluents are reused for irrigation, infiltration, sprinkling, and vial wash-down. (o.7.3) Provide treatment waste services to third parties. (o.7.4) Treatment of effluents with average daily pollutant load ≥ the equivalent sewage for 100 inhabitants, in one or more of the discharge quality standards.
o.8	Treatment and/or disposal systems for solid industrial waste with treatment capacity ≥30 tons per day or ≥50 tons of disposal.
o.9	Treatment and/or disposal and/or elimination systems for hazardous waste with treatment capacity of 1000 kg per day and 25 kg per day for those catalogued as “acute toxic” waste according to the Supreme Decree SD148 of 2003 of the Ministry of Health [23].
o.10	Treatment and/or disposal and/or elimination systems for special hazardous waste from human health requirements, with treatment capacity ≥250 kg per day.
o.11	Repair or recovery of contaminated areas covering ≥1 hectare.

**Table 2:** Selected EIS and EID sanitation projects to implement the statistical analysis, catalogued by macrozone and region of Chile.

Macrozone	Region	Way for Projects’ Entry	
		EIS	EID
North Zone	XV, Arica and Parinacota	1	2
	I, Tarapacá	1	2
	II, Antofagasta	1	2
	III, Atacama	2	1
	IV, Coquimbo		3
Central Zone	V, Valparaíso	1	3
	RM, Metropolitan	2	7
	VI, O’Higgins		4
	VII, Maule	1	9
South Zone	XVI, Ñuble	1	2
	VIII, Bio Bio	1	4
	IX, La Araucanía	1	3
	XIV, Los Ríos	1	1
	X, Los Lagos	1	10
Austral Zone	XI, Aysén	1	6
	XII, Magallanes		2
		15	61

**Table 3:** Selected indicators to compare samples of sanitation projects.

Indicator	Description	Options	Score	References
A	Processing time (working days)	≥361	1	[36]
		271–360	2	
		181–270	3	
		91–180	4	
		1–90	5	
B	Description of the influence area	No information	1	[36]
		Information not justified	2	
		General information only	3	
		Moderately justified information	4	
		Detailed and justified information	5	
C	Methodology to identify and evaluate environmental impacts	Yes	2	[29,36]
		No	1	
D	Use of reference international regulations	Yes	2	[29,36]
		No	1	
E	Existence of mitigation measures	Yes	2	[26,36]
		No	1	
F	Existence of compensation measures	Yes	2	[26,36]
		No	1	
G	Existence of repair measures	Yes	2	[26,36]
		No	1	
H	Identification of contingency and emergency measures	Yes	2	[26,36]
		No	1	
I	Consultation and participation	Yes	2	[24,26,27,36]
		No	1	
J	Appeal after project approval or rejection	Yes	2	[26,36]
		No	1	
K	Public information after the environmental license is obtained	Yes	2	[32]
		No	1	
L	Post-auditing	No information about supervision or Unsupervised project	1	[28,29]
		Project without non-compliance	2	
		Breach of the environmental license or Sector Permits	3	
M	Punishment for non-compliance	No information	1	[28,29]
		Project without infraction	2	
		No classified or minor infraction	3	
		Serious infraction	4	
		Very serious infraction	5	
N	Investment (MUSD)	0–0.5	1	–
		>0.5–1	2	
		>1–10	3	
		>10–100	4	
		>100	5	

## Data Availability

Data are included in Appendix A.

## Appendix A

**Table A1:** Compiled sanitation projects by region entered as EIS and EID in the Chilean EIA System. Information available at the EAS website (www.sea.gob.cl) (accessed on 11 January 2021).

Region	Approved		Rejected		In Evaluation		Unadmitted		No Rated		Abandoned		Desisted		License Expired		Revoked	
	EIS	EID	EIS	EID	EIS	EID	EIS	EID	EIS	EID	EIS	EID	EIS	EID	EIS	EID	EIS	EID
XV, Arica and Parinacota	1	28			1			12	1				1	7				1
I, Tarapacá	2	54	1	4				21	1			1		13				
II, Antofagasta	8	123		3			1	38		3	1		3	17		1		
III, Atacama	6	87	2				2	29		1			2	10				
IV, Coquimbo	4	87	1	3			4	56		1			2	29				
V, Valparaíso	14	166	6	9			5	80	1	4	1	6	4	50				
RM, Metropolitan	25	414	15	20				176	1	20	1	4	12	173		2		1
VI, O’Higgins	2	267		20				73		5		4	4	71				2
VII, Maule	7	480	1	28				61	1	2			4	90		2		
XVI, Ñuble	5	100	3	6			2	29	1	3			4	24				1
VIII, Bio Bio	14	223	2	6			3	70		1			8	53				
IX, La Araucanía	8	146	2	11	2		3	29		1		1	4	27				
XIV, Los Ríos	7	83		8	1	1	1	19		6			3	44				2
X, Los Lagos	9	536	1	46				53		13		3		86		2		1
XI, Aysén	4	316	1	15			1	36		1	1		3	43		1		
XII, Magallanes	3	100	1	6				22		4			1	24				
Interregional	1	9		1				11					2	5				
Total	120	3219	36	186	4	1	22	815	6	65	4	19	57	766	0	8	0	8
